# The Association between Migraine and Abdominal Aortic Aneurysms: A Nationwide Population-Based Cohort Study

**DOI:** 10.3390/ijerph18084389

**Published:** 2021-04-20

**Authors:** Jou-Yu Lin, Che-Se Tung, Jen-Chun Wang, Wu-Chien Chien, Chi-Hsiang Chung, Chih-Yuan Lin, Shih-Hung Tsai

**Affiliations:** 1 Department of Rehabilitation, Cheng Hsin General Hospital, Taipei 11220, Taiwan; squidurreal@hotmail.com; 2 Division of Medical Research & Education, Cheng Hsin General Hospital, Taipei 11220, Taiwan; ch8388@chgh.org.tw; 3 Department of Emergency Medicine, Tri-Service General Hospital, National Defense Medical Center, Taipei 11490, Taiwan; royalflushwang@gmail.com; 4 Department of Medical Research, Tri-Service General Hospital, National Defense Medical Center, Taipei 11490, Taiwan; g694810042@gmail.com; 5 School of Public Health, National Defense Medical Center, Taipei 11490, Taiwan; 6 Taiwanese Injury Prevention and Safety Promotion Association, Taipei 11490, Taiwan; 7 Department of Surgery, Division of Cardiovascular Surgery, Tri-Service General Hospital, National Defense Medical Center, Taipei 11490, Taiwan; linrock@ms26.hinet.net; 8 Department of Physiology and Biophysics, Graduate Institute of Physiology, National Defense Medical Center, Taipei 11490, Taiwan

**Keywords:** abdominal aortic aneurysm, migraine, cardiovascular diseases, National Health Insurance Research Database

## Abstract

Previous studies have indicated that patients with migraine have a higher prevalence of risk factors known to be associated with cardiovascular diseases. There are also shared epidemiology and molecular mechanisms between migraine and abdominal aortic aneurysm (AAA). We hypothesized that patients with migraine could have an increased risk of AAA. To test this hypothesis, we used the National Health Insurance Research Database (NHIRD) to evaluate whether associations exist between migraine and AAA. The data for this nationwide population-based retrospective cohort study were obtained from the NHIRD in Taiwan. The assessed study outcome was the cumulative incidence of AAA in patients with migraine during a 15-year follow-up period. Among the 1,936,512 patients from the NHIRD, 53,668 (2.77%) patients were identified as having been diagnosed with migraine. The patients with migraine had a significantly higher cumulative risk of 3.558 of developing an AAA 5 years after the index date compared with the patients without migraine. At the end of the 15-year follow-up period, a significantly higher incidence of AAA (0.98%) was observed in the patients with migraine than in those without migraine (0.24%). We revealed an association between the development of migraine and AAA.

## 1. Introduction

Migraine affects as many as 25% of women by their mid-to-late 30s, nearly 15% of the population and approximately one billion people worldwide [1]. Previous studies have indicated that patients with migraine have an increased risk of several intracranial pathologies, such as stroke, subcortical small vessel diseases and increased carotid intimal thickness [2,3,4]. Patients with migraine have a higher prevalence of risk factors known to be associated with cardiovascular diseases (CVDs), including hypertension, hyperlipidemia and smoking [5,6,7,8].

There are risk factors and epidemiological and molecular mechanisms that are shared between migraine and abdominal aortic aneurysm (AAA). Hypertension, hyperlipidemia and smoking are risk factors for both AAA and migraine [9,10]. Activated matrix metalloproteinases (MMPs), endothelial dysfunction and vascular inflammation also overlap in the disease pathogenesis of AAA and migraine [11,12,13,14]. However, there has been limited research regarding the association between migraine and AAA. We hypothesized that patients with migraine could have an increased risk of AAA.

For the purpose of testing this hypothesis, we used the National Health Insurance Research Database (NHIRD) to evaluate whether associations exist between migraine and AAA.

## 2. Materials and Methods

### 2.1. Data Source

The National Health Insurance (NHI) Program includes more than 99% of the entire Taiwanese population (more than 23 million beneficiaries in 2018); the NHI was launched in Taiwan in 1995. The NHIRD contains encrypted patient identification numbers, birthdays, sexes, dates of each admission and discharge, ICD-9-CM (International Classification of Diseases, 9th Revision, Clinical Modification) diagnostic (up to five each) and procedure codes, medications and outcomes. The data we used were obtained from a subset of the NHIRD named the Longitudinal Health Insurance Database 2005 (LHID 2005). The LHID 2005 contains information on each medical service utilization for approximately two million beneficiaries who were randomly selected from the NHIRD. The NHI Administration randomly and periodically reviews the medical records to verify the accuracy of the diagnoses. The accuracy of the diagnoses of major diseases in the NHIRD, such as aortic aneurysm, aortic dissection, migraine, acute coronary syndrome and stroke, has been validated in previous studies [15,16,17,18,19].

### 2.2. Sampled Patients

Study and comparison cohorts were included. We tracked individual patients in this study. Patients in the LHID 2005 database aged ≥ 20 years who were newly diagnosed with migraine (ICD-9-CM 346.0–346.1) were selected and followed up between 2000 and 2015. The date of the diagnosis of migraine was used as the index date. We excluded patients who had been diagnosed with migraines or had an AAA prior to the index date, who were aged < 20 years and who had a follow-up duration of less than 6 months. Control patients were selected from individuals in the LHID 2005. The patient and control cohorts were selected by 1:4 matching according to age, sex, comorbidities, including hypertension (ICD-9-CM 401–405), diabetes mellitus (ICD-9-CM 250), hyperlipidemia (ICD-9-CM 272.0–272.4), acute ischemic stroke (AIS; ICD-9-CM 433–434, 436, 437.1), intracerebral hemorrhage (ICH; ICD-9-CM 430, 431, 432.9), coronary artery disease (CAD; ICD-9-CM 410–414), atrial fibrillation (AF; ICD-9-CM 427.31), heart failure (HF; ICD-9-CM 428), chronic obstructive pulmonary disease (COPD; ICD-9-CM 490–496), chronic kidney disease (CKD; ICD-9-CM 580–589) and cancer (ICD-9-CM 140–208) and the number of medical visits. The long-term medications, according to the Anatomical Therapeutic Chemical (ATC) codes defined by the World Health Organization (WHO), thought to be associated with the treatment of migraine or AAA were recorded; these included antihypertensive agents, beta-blockers, calcium channel blockers, diuretics and angiotensin-converting enzyme inhibitors or angiotensin II receptor blockers; statins; selective serotonin agonists; antimigraine agents, including ergot alkaloids, topiramate, valproate, tricyclic antidepressants, selective serotonin reuptake inhibitors and flunarizine; nonsteroidal anti-inflammatory drugs (NSAIDs) and analgesic drugs other than NSAIDs, including aspirin, acetaminophen and cyclooxygenase-2 inhibitors. The incidence of AAA (ICD-9-CM 441.3–441.7) after 2005 was the outcome.

### 2.3. Patient and Public Involvement

Neither the patients nor the public was involved in the design or planning of this study.

### 2.4. Statistical Analysis

All statistical analyses were performed using SPSS software, version 22 (SPSS Inc., Chicago, IL, USA). The clinical characteristics of the patients enrolled in the study are expressed in numerical form. Categorical variables, which are presented as percentages, were compared using Fisher’s exact test and chi-square tests. Continuous variables are presented as the means and standard deviations and were compared using *t*-tests. The primary goal of the study was to determine whether the clinical characteristics of the patients were associated with the development of AAA. Fine and Gray’s competing risk analysis was used to determine the risk of migraine-related morbidities, as death can act as a competing risk factor for AAA. The associations between time-to-event outcomes (prognoses) and clinical characteristics were examined using the Kaplan–Meier method and multivariate Cox regression analysis. Multivariate Cox regression analysis with stepwise selection was selected to avoid possible collinearity. This process adjusted all the variables mentioned above. The results are presented as adjusted hazard ratios (HRs) with the corresponding 95% confidence intervals (CIs). Statistical significance was indicated by *p* < 0.05.

## 3. Results

**Figure 1:**
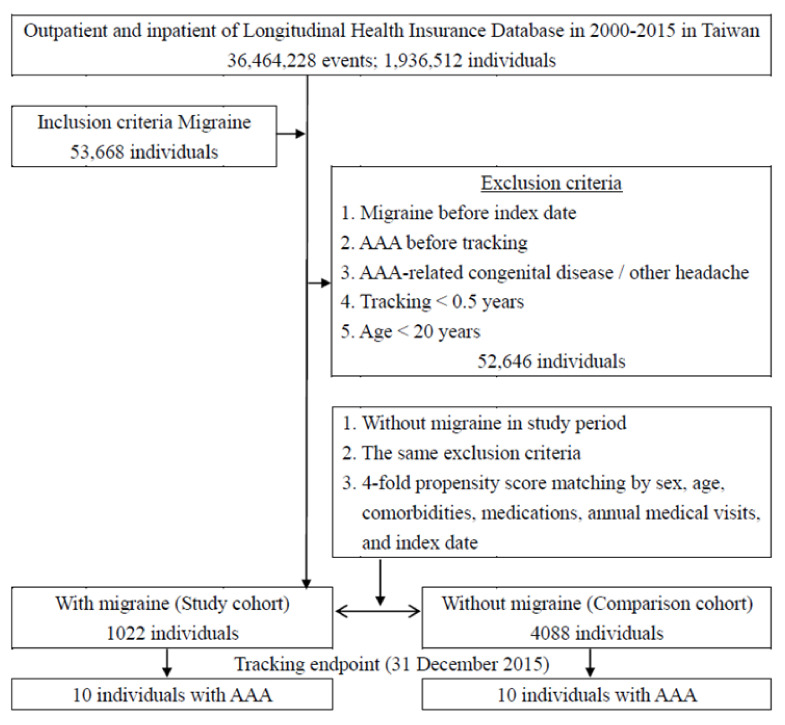
Patient selection flowchart.

Among the 1,936,512 patients in the LHID 2005–2015 from the NHIRD, 53,668 patients were identified as having been diagnosed with migraine. In total, 1022 patients were then assigned to the study cohort and another 4088 age-, sex- and comorbidity-matched patients formed the comparison cohort (Figure 1). There were no significant differences in sex, age, comorbidities or the number of medical follow-up visits between the groups with and without migraine after matching (Table 1). The estimated power of this study is 0.78. The Omnibus test for Cox regression was significant (*p* < 0.001).

**Figure 2:**
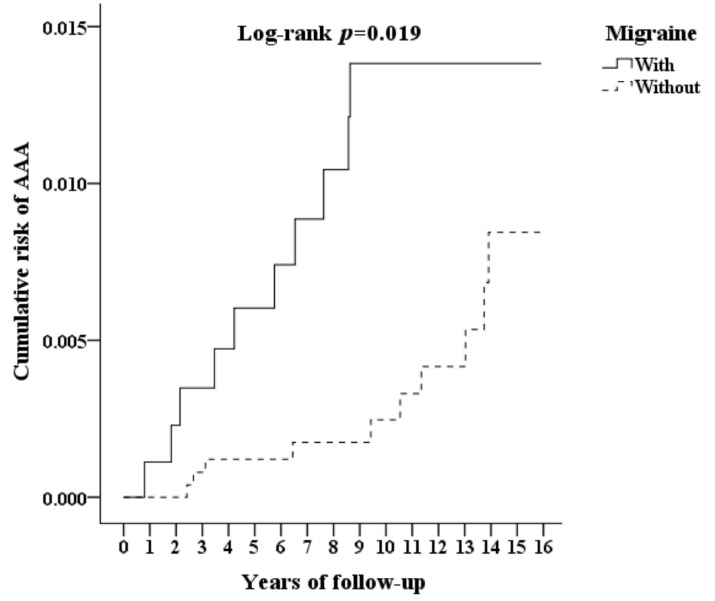
Kaplan–Meier curve for the cumulative risk of abdominal aortic aneurysm due to migraine.

**Figure 3:**
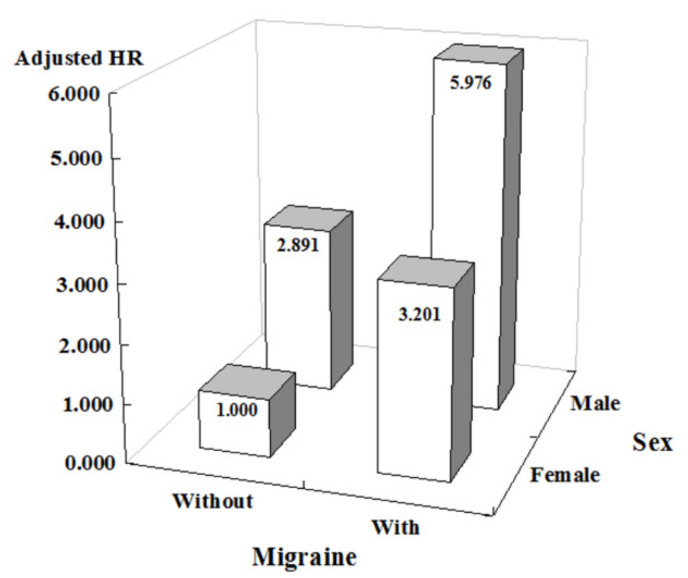
Interaction model of the risk of AAA due to male sex and migraine.

The patients who had migraine had a significantly higher cumulative risk of developing AAA 5 years after the index date compared with the patients without migraine (log-rank test *p* = 0.019, Figure 2). At the end of the 15-year follow-up period, a significantly higher incidence of AAA (0.98% vs. 0.24%, *p* = 0.003) and several comorbidities, including hypertension, hyperlipidemia, COPD and malignancies, were observed in the patients with migraine than in those without migraine (Table 2). The patients with migraine also exhibited a significantly increased incidence of AAA compared with patients without migraine, according to Cox regression analysis and Fine and Gray’s competing risk model (adjusted HR = 3.558, 95% CI = 1.439–8.799, *p* = 0.006, Table 3). In addition, male sex (adjusted HR = 3.008, 95% CI = 1.132–7.998, *p* = 0.027), ICH (adjusted HR = 22.406, 95% CI = 4.476–112.235, *p* < 0.001) and CAD (adjusted HR = 4.402, 95% CI = 1.189–13.738, *p* < 0.001) were also associated with an increased risk of developing AAA. There were no differences regarding the use of cardiovascular medications, antimigraine medications, NSAIDs or flunarizine at the end of the study. Because the most prominent risk factors for AAA were male sex and migraine, an interaction model was employed. The results showed that males with migraine had a 5.976-fold (95% CI 2.409–15.763, *p* = 0.001) increased risk of AAA compared with females without migraine (Figure 3). In addition, patients with migraine with aura (MA) were more likely to develop AAA than patients who had migraine without aura (MO) (Table 4).

## 4. Discussion

In this population-based study of a nationwide dataset, we revealed an association between migraine and AAA, even after adjusting for age, sex, comorbidities and annual medical follow-ups. Estimates of the prevalence of chronic migraine throughout the world are up to 5% [20]. Baykan’s study indicated that migraine incidence was estimated as 2.38% (2.98% in women and 1.93% in men) per year in 2563 migraine-free individuals in Turkey [21]. Previous studies from Taiwan reported that the population prevalence of chronic migraine ranges from 1.0% to 1.7% [22,23]. Our study is a longitudinal study with a 15-year follow-up. Here we identified 53,668 migraine patients in 1,936,512 individuals (2.77%) over 15 years. A previous population-based nationwide sample that used International Classification of Headache Disorders criteria with structured headache interviews showed a 2.38% incidence rate of migraine [21]. In a recent Swedish study involving ultrasonographic screening of 65-year-old men, the prevalence of AAA was 2.2%, whereas, in earlier studies, the reported prevalence was as high as 8% among men 65 to 80 years of age [9,24,25]. There are shared risk factors for AAA and migraine, including hypertension and hyperlipidemia [5], while diabetes appears to be a protective factor for both migraine and AAA [26,27]. Studies have indicated associations between migraine and intracranial pathologies, such as an increased risk of AIS, ICH, subcortical small vessel diseases, white matter lesions and even epilepsy [2,3,4]. In a population-based study of stroke etiology stratified by age, migraine was strongly associated with AIS, suggesting a causal role or a shared etiology [28]. Shared genetic mutations and inflammatory, vascular, endothelial and coagulable factors have been suggested as putative mechanisms for both migraine and stroke [29]. A previous study indicated an association between AAA and AF [30]. Patients with migraine also had an increased risk of AF [31,32]. Migraine prevalence is increased in patients with intracranial aneurysms (ICAs) [33,34]. Our previous study revealed an association between ICAs and aortic aneurysms, and open surgical repair was associated with fewer recognized ICAs than nonsurgical treatment [35]. Patients with aortic aneurysm and aortic dissection also had an increased risk of subarachnoid hemorrhage (SAH) [36]. In this study, we also identified ICH as being linked to the development of AAA.

In addition to the associations between migraine and cerebrovascular diseases, migraine has been associated with atherosclerotic vascular diseases, such as CADs, myocardial infarction and increased risk of carotid thickness [32,37,38,39,40]. Patients with migraine have also been reported to have a higher frequency of cardiovascular risk factors, including hypertension and hyperlipidemia [7,8,41]. Smoking is a precipitating factor for migraine attacks [6]. A previous study revealed an increase in the onset of CVD within the first year of a migraine diagnosis [32]. Previous studies have also indicated that MA, but not MO, is associated with an increased risk of carotid thickening and elevated vascular biomarkers [12,37]. Consistently, we also found that patients with MA are more likely to develop AAA than patients who have MO. There are overlapping risk factors and underlying molecular mechanisms for both migraine and AAA. The reported risk factors for the development of AAA have been age, male sex, smoking, hypertension, high body mass index, hyperlipidemia and family history [9,10]. Hypertension, hyperlipidemia and smoking have also been reported as risk factors for migraine [5]. The mechanisms by which migraine might increase AAA are probably multifactorial. A proinflammatory state has also been suggested in migraine. Both migraine and AAA are associated with the overexpression of MMPs, endothelial activation and vascular inflammation. Reactive oxygen species are generated by endothelial cells during migraine attacks [11,12,13]. Consistent with previous studies, we also identified CAD and malignancy as risk factors for AAA development [42,43,44]. A previous study demonstrated an association between spontaneous coronary artery dissection (SCAD) and migraine. At the time of SCAD, patients with migraine were younger than those without migraine and had more aneurysms, pseudoaneurysms and dissections based on coronary angiograms [45].

The use of NSAIDs might increase the risk of CVDs, such as AMI, HF and AF [46,47,48]. In this study, we found that medications were not associated with an increased incidence of AAA in patients with migraine. No clinical studies have revealed that commonly prescribed cardiovascular medications, including statins, angiotensin-converting enzyme inhibitors, angiotensin receptor blockers, beta-blockers and calcium channel blockers, can effectively limit the progression of AAA [49]. Surgical intervention is required to prevent rupture in patients who have an AAA growth rate exceeding 0.5 cm in diameter over a period of 6 months and an aortic diameter of 5.5 cm in men and 5.0 cm in women [9,50]. The risk of AAA rupture is determined by the size of the aneurysm; rupture occurs in approximately 2% of AAAs less than 4 cm in diameter and in more than 25% of AAAs larger than 5 cm [51]. Although being a younger female is a risk factor for migraine, we found that being male with migraine had a nearly six-fold increased risk of AAA development. We, therefore, propose that male patients with migraine should undergo additional screening examinations for AAA.

### Limitations

This study has several limitations. The NHIRD registry does not provide detailed information regarding family histories, health-related lifestyle factors, quality of life or imaging and laboratory results, which may represent potential confounding factors. Cigarette smoking is a risk factor for AAA, but these data are not provided by the NHIRD. We used the incidence of COPD as a proxy variable for tobacco use to account for the potential confounding effect of tobacco use in our study design [52]. We utilized multivariate logistic regression models to adjust our results. The diagnostic criteria are based on clinical symptoms; therefore, the accuracy of the diagnosis of migraine or aura is difficult to validate. Detailed information regarding the exact diameters of the AAAs was unavailable in the NHIRD.

## 5. Conclusions

We observed an association between migraine, particularly MA, and the development of AAA even after adjusting for several comorbidities and medications in a nationwide population database.

## Figures and Tables

**Table 1:** Characteristics of the study participants at baseline.

	Total	With Migraine	Without Migraine	*p*-Value
	*N* (%)	*N* (%)	*N* (%)	
Total	5110	1022 (20%)	4088 (80%)	
Sex				0.999
Male	1640 (32.09%)	328 (32.09%)	1312 (32.09%)	
Female	3470 (67.91%)	694 (67.91%)	2776 (67.91%)	
Age (years)	47.12 ± 16.86	46.92 ± 16.46	47.17 ± 16.96	0.674
Hypertension	488 (9.55%)	109 (10.67%)	379 (9.27%)	0.190
Hyperlipidemia	180 (3.52%)	40 (3.91%)	140 (3.42%)	0.448
DM	390 (7.63%)	64 (6.26%)	326 (7.97%)	0.065
Ischemic stroke	112 (2.19%)	22 (2.15%)	90 (2.20%)	0.924
Intracerebral hemorrhage	25 (0.49%)	6 (0.59%)	19 (0.46%)	0.617
CAD	298 (5.83%)	72 (7.05%)	226 (5.53%)	0.073
AF	39 (0.76%)	4 (0.39%)	35 (0.86%)	0.159
HF	75 (1.47%)	14 (1.37%)	61 (1.49%)	0.885
COPD	462 (9.04%)	88 (8.61%)	374 (9.15%)	0.592
CKD	137 (2.68%)	23 (2.25%)	114 (2.79%)	0.387
Malignancy	118 (2.31%)	21 (2.05%)	97 (2.37%)	0.545
Annual medical visiting	7.82 ± 6.83	7.65 ± 6.79	7.86 ± 6.84	0.379

*p*-value (category variable: Chi-square/Fisher exact test; continue variable: *t*-test); AF: atrial fibrillation; DM: diabetes mellitus; HF: heart failure; CAD: coronary artery disease; CKD: chronic kidney disease; COPD: chronic obstructive pulmonary disease.

**Table 2:** Incidence rates of abdominal aortic aneurysm and other characteristics in the enrolled study participants at the end of the 15-year follow-up period.

	Total	With Migraine	Without Migraine	*p*-Value
	*N* (%)	*N* (%)	*N* (%)	
Total	5110	1022 (20.00%)	4088 (80.00%)	
AAA	20 (0.39%)	10 (0.98%)	10 (0.24%)	0.003 *
Sex				0.999
Male	1640 (32.09%)	328 (32.09%)	1312 (32.09%)	
Female	3470 (67.91%)	694(67.91%)	2776 (67.91%)	
Age (years)	54.02 ± 18.39	56.02 ± 17.70	53.52 ± 18.53	<0.001 *
Hypertension	797 (15.60%)	200 (19.57%)	597 (14.60%)	<0.001 *
DM	643 (12.58%)	147 (14.38%)	496 (12.13%)	0.058
Hyperlipidemia	121 (2.37%)	37 (3.62%)	84 (2.05%)	0.005 *
Ischemic stroke	130 (2.54%)	30 (2.94%)	100 (2.45%)	0.375
Intracerebral hemorrhage	52 (1.02%)	14 (1.37%)	38 (0.93%)	0.222
CAD	336(6.58%)	80 (7.83%)	256 (6.26%)	0.078
AF	71(1.39%)	14 (1.37%)	57 (1.39%)	0.952
HF	176 (3.44%)	36 (3.52%)	140 (3.42%)	0.848
COPD	310 (6.07%)	81 (7.93%)	229 (5.60%)	0.007 *
CKD	287 (5.62%)	56 (5.48%)	231 (5.65%)	0.879
Malignancy	540 (9.86%)	74 (7.24%)	430 (10.52%)	0.002 *

* *p*-values < 0.05 were considered significant. AAA = abdominal aortic aneurysm; AF = atrial fibrillation; DM = diabetes mellitus; HF = heart failure; CAD = coronary artery disease; CKD = chronic kidney disease; COPD = chronic obstructive pulmonary disease.

**Table 3:** Factors associated with abdominal aortic aneurysms according to the Cox regression model.

Variables	Crude HR	95% CI		*p*	Adjusted HR	95% CI		*p*-Value
Migraine	2.757	1.146	6.632	0.024	3.558	1.439	8.799	0.006 *
Male	3.192	1.301	7.833	0.011	3.008	1.132	7.998	0.027 *
Age (years)	1.011	0.983	1.039	0.452	0.993	0.961	1.026	0.684
Hypertension	1.012	0.149	1.754	0.287	1.422	0.115	1.550	0.194
DM	1.504	0.117	2.174	0.358	1.487	0.108	2.209	0.351
Hyperlipidemia								
Ischemic stroke	4.266	0.989	18.406	0.052	4.462	0.952	20.900	0.058
Intracerebral hemorrhage	13.009	2.968	57.021	0.001	22.406	4.476	112.235	<0.001 *
CAD	2.757	0.921	8.252	0.070	4.402	1.189	13.738	0.025 *
AF								
HF	2.962	0.678	12.941	0.149	1.985	0.388	10.144	0.410
COPD	2.245	0.656	7.679	0.198	1.897	0.501	7.176	0.346
CKD	1.934	0.123	7.100	0.948	1.662	0.084	5.214	0.695
Malignancy	2.550	0.841	7.732	0.098	3.848	1.190	12.438	0.024 *

* *p*-values < 0.05 were considered significant. AAA = abdominal aortic aneurysm; AF = atrial fibrillation; DM = diabetes mellitus; HF = heart failure; CAD = coronary artery disease; CKD = chronic kidney disease; COPD = chronic obstructive pulmonary disease.

**Table 4:** Sensitivity test for factors of AAA and using Cox regression and Fine and Gray’s competing risk model.

		Competing Risk in the Model				
Sensitivity Test	Migraine Subgroups	Adjusted HR		95% CI		*p*-Value
Overall	Without migraine	Reference				
	With migraine	3.558		1.439	8.799	0.006
	With aura	5.426		2.201	16.984	<0.001
	Without aura	1.502		0.906	2.978	0.211

## Data Availability

Restrictions apply to the availability of these data. Data was obtained from Taiwan NHIRD and are available under request with the permission of NHIRD.
